# Caloric restriction and metformin selectively improved LKB1-mutated NSCLC tumor response to chemo- and chemo-immunotherapy

**DOI:** 10.1186/s13046-023-02933-5

**Published:** 2024-01-02

**Authors:** Gloriana Ndembe, Ilenia Intini, Massimo Moro, Chiara Grasselli, Andrea Panfili, Nicolò Panini, Augusto Bleve, Mario Occhipinti, Cristina Borzi, Marina Chiara Garassino, Mirko Marabese, Simone Canesi, Eugenio Scanziani, Gabriella Sozzi, Massimo Broggini, Monica Ganzinelli

**Affiliations:** 1 Laboratory of Molecular Pharmacology, Department of Experimental Oncology, Istituto Di Ricerche Farmacologiche Mario Negri IRCCS, Milan, Italy; 2 Tumor Genomics Unit, Department of Experimental Oncology and Molecular Medicine, Fondazione IRCCS Istituto Nazionale Dei Tumori, Milan, Italy; 3 Immunopharmacology Unit, Department of Experimental Oncology, Istituto Di Ricerche Farmacologiche Mario Negri IRCCS, Milan, Italy; 4 Thoracic Unit, Medical Oncology Department, Fondazione IRCCS Istituto Nazionale Dei Tumori, Milan, Italy; 5 Mouse & Animal Pathology Lab, Fondazione Filarete, Milan, Italy; 6 Department of Veterinary Medicine, University of Milan, Milan, Italy

**Keywords:** NSCLC, KRAS, LKB1, Cancer metabolism, Metformin, Caloric restriction

## Abstract

**Background:**

About 10% of NSCLCs are mutated in *KRAS* and impaired in *STK11/LKB1*, a genetic background associated with poor prognosis, caused by an increase in metastatic burden and resistance to standard therapy. LKB1 is a protein involved in a number of biological processes and is particularly important for its role in the regulation of cell metabolism. *LKB1* alterations lead to protein loss that causes mitochondria and metabolic dysfunction that makes cells unable to respond to metabolic stress. Different studies have shown how it is possible to interfere with cancer metabolism using metformin and caloric restriction (CR) and both modify the tumor microenvironment (TME), stimulating the switch from “cold” to “hot”.

Given the poor therapeutic response of *KRAS*^mut^/*LKB1*^mut^ patients, and the role of LKB1 in cell metabolism, we examined whether the addition of metformin and CR enhanced the response to chemo or chemo-immunotherapy in *LKB1* impaired tumors.

**Methods:**

Mouse cell lines were derived from lung nodules of transgenic mice carrying KRAS^G12D^ with either functional LKB1 (KRAS^G12D^/LKB1^wt^) or mutated LKB1 (KRAS^G12D^/LKB1^mut^). Once stabilized in vitro, these cell lines were inoculated subcutaneously and intramuscularly into immunocompetent mice. Additionally, a patient-derived xenograft (PDX) model was established by directly implanting tumor fragments from patient into immunocompromised mice.

The mice bearing these tumor models were subjected to treatment with chemotherapy or chemo-immunotherapy, both as standalone regimens and in combination with metformin and CR.

**Results:**

Our preclinical results indicate that in NSCLC *KRAS*^mut^/*LKB1*^mut^ tumors, metformin and CR do enhance the response to chemo and chemo-immunotherapy, inducing a metabolic stress condition that these tumors are not able to overcome. Analysis of immune infiltrating cells did not bring to light any strong correlation between the TME immune-modulation and the tumor response to metformin and CR.

**Conclusion:**

Our in vitro and in vivo preliminary studies confirm our hypothesis that the addition of metformin and CR is able to improve the antitumor activity of chemo and chemoimmunotherapy in LKB1 impaired tumors, exploiting their inability to overcome metabolic stress.

## Background

Lung cancer is one of the leading causes of cancer death worldwide, with more than two million new cases and 1.8 million deaths per year [1, 2]. Non-small-cell lung cancer (NSCLC) is the most frequent (nearly 85%) with a five-year survival of about 25% when all stages are considered [3, 4]. The development of targeted therapies and the introduction of immunotherapy have reinvented the treatment of advanced-stage NSCLC, leading to impressive gains in survival [5–9]. However, the heterogeneity of this pathology means that a substantial subgroup of patients does not benefit from the current treatment lines [10, 11].

Liver kinase B1 (*LKB1*, also known as Serine/threonine kinase 11, STK11), responsible for maintenance of the balance between anabolic and catabolic processes, is mutated in about 30% of all adenocarcinoma and the co-mutation with *KRAS* is often reported [12–14]. The co-occurrence of these mutations is associated with poor prognosis, related to a particularly aggressive cancer phenotype resistant to chemo-immunotherapy, for which no targeted therapies are available [15–17].

Almost invariably, *LKB1* alterations lead to protein loss that causes mitochondria and metabolic dysfunction that make cells unable to respond to metabolic stress [18, 19], a vulnerability that can be exploited to selectively target these tumors. Various studies, conducted also by our group, have shown that a lack of LKB1 sensitizes adenocarcinoma cells to metabolic stress induced by compounds that lower intracellular energy levels, or nutrient and growth factor deprivation, resulting in metabolic crisis and apoptosis [14, 20, 21].

Metformin, widely used as first-line therapy in type 2 diabetes, has attracted attention for its anticancer activity related to both its cellular and systemic effects [22, 23]. At cellular level, metformin inhibits complex I of the mitochondrial electron transport chain, lowering ATP cellular levels, increasing the AMP/ATP ratio, and inducing the switch from anabolic to catabolic metabolism. At systemic level metformin lowers circulating glucose and insulin, the mechanism likely related to decreases of hormones, cytokines and metabolic intermediates associated with tumorigenesis, tumor growth and progression [24]. Metformin can also prevent acquired resistance to cisplatin reducing the number of cancer stem cells [20] and increasing the immunotherapy response, boosting tumor cell immune sensitivity to T cells [25].

Given its anticancer activity and safety profile, multiple clinical trials are investigating the adjuvant effect of metformin in combination with other treatments.

There is increasing evidence of the possibility of affecting tumor metabolism using restrictive dietary approaches [26]. Different strategies have been developed, including caloric restriction (CR), intermittent fasting and ‘fasting-mimicking diet’ (FMD), to reduce the availability of essential metabolites vital for various important pathways related to cancer cell growth and dissemination [27].

Several studies have highlighted the synergistic anticancer effect of the combination of these dietary approaches with cytotoxic agents, as well as their protective action on normal cells from treatment-induced adverse effects [26, 28, 29]. Dietary intervention is also involved in the modification of the tumor microenvironment (TME), stimulating the recruitment of CD8^+^ lymphocytes that lead to an inflamed TME which can be exploited to sensitize tumors to immunotherapy [30, 31].

Considering the difficulties in the clinical use of fasting due to complications like weight loss, cachexia, fatigue and nausea [32], FMD is likely to be better tolerated, being less restrictive and providing the body’s essential nutrients while maintaining the beneficial effects of fasting [33].

Targeting one specific metabolic pathway may not be sufficient to induce cancer cell death, because of the metabolic plasticity of cancer cells. The present study therefore investigated the effect of metabolic stress caused by the combination of metformin and caloric restriction on the response to chemo and immunotherapy.

## Materials and methods

### Cell lines and cell viability assay

Mouse cell lines were generated from lung nodules of KRAS^G12D^/LKB1^wt^ (K) and *KRAS*^G12D^/*LKB1*^del^ (KL) transgenic mice as described in [34], and cultured in Roswell Park Memorial Institute (RPMI)-1640 supplemented with 2 mM of L-glutamine (Microgem) and 10% (v/v) fetal bovine serum (FBS, Euroclone). Cells were grown at 37°C in a humidified atmosphere supplemented with 5% (v/v) CO_2_.

For cytotoxicity experiments, K and KL cell lines were seeded at respectively 6500 c/mL and 7000 c/mL, in 96-well plates and treated after 24 h with 2 mM of metformin (1,1-Dimethylbiguanide hydrochloride, #D150959 Sigma-Aldrich) and a 50% reduction of glucose, glutamine and FBS in the medium. Cell viability was examined after 72 h with MTS assay (Promega) and absorbance was acquired using a plate reader (GloMax Discover, Promega). The mean and SD of at least three independent experiments, each consisting of six replicates, are presented.

The cell lines are routinely tested by polymerase chain reaction for mycoplasma contamination.

### Seahorse flux analysis

The fractions of ATP produced from mitochondrial oxidative phosphorylation (OXPHOS) and glycolysis were measured using a Seahorse XFe24 analyzer XF Real-Time ATP Rate Assay Kit (Agilent). Briefly, K and KL cell lines were seeded at respectively 7500 and 8000 cells/well in poly-L-lysine coated 96-well Sea-horse plates (Agilent). After 12 h cells were treated with 2mM metformin, 50% CR or the combination. After 24 h the cells were washed twice with Seahorse XF DMEM Medium (pH 7.4, 10 mM of XF glucose, 1 mM of XF pyruvate and 2 mM of glutamine) and incubated one hour with the same medium at 37°C without CO_2_. The oxygen consumption rate (OCR) and extracellular acidification rate (EACR) were measured at baseline and after a serial injection of 1.5 μM oligomycin (ATP synthase inhibitor) and a mix of 0.5 μM rotenone (mitochondrial complex I inhibitor) and 0.5 μM antimycin A (mitochondrial complex II inhibitor) to determine real-time metabolic changes. All ATP rates were normalized to total protein content and the treated cells were compared to untreated ones. ATP production rates were calculated as described by Wang et al. [35].

### Protein extraction and Western blotting

Proteins were extracted and visualized as reported in [36]. Immunoblotting was carried out with the following antibodies: anti-phospho-p44/42 MAPK (ERK 1/2) (Thr202/Tyr204) #9101S, anti-p44/42 MAPK (ERK1/2) #9102S, anti-phospho-S6 Ribosomal Protein (Ser235/236) #221 and anti-S6 Ribosomal protein #2217 purchased from Cell Signaling Technology. Anti-RAN #sc-271376 was purchased from Santa Cruz Biotechnology. The secondary antibodies anti-rabbit #170–6515 and anti-mouse #170–6516 from Bio Rad were used.

### Mouse tumor models and in vivo treatments

Five-week-old female C57BL/6 mice, purchased from Charles River Laboratories Italia S.p.a were used.

All animals were housed at constant temperature and humidity, according to institutional guide-lines and maintained under standard pathogen-free conditions. The Istituto di Ricerche Farmacologiche Mario Negri IRCCS adheres to national and international laws, regulation and policies on the maintenance, care and use of laboratory animals.

Mice were injected subcutaneously in the flank or intramuscularly in the gastrocnemius with 5 × 10^5^ and 10^5^ K or KL cells, respectively, and randomized in different groups. PDXs were established as described [37].

**Fig. 1:**
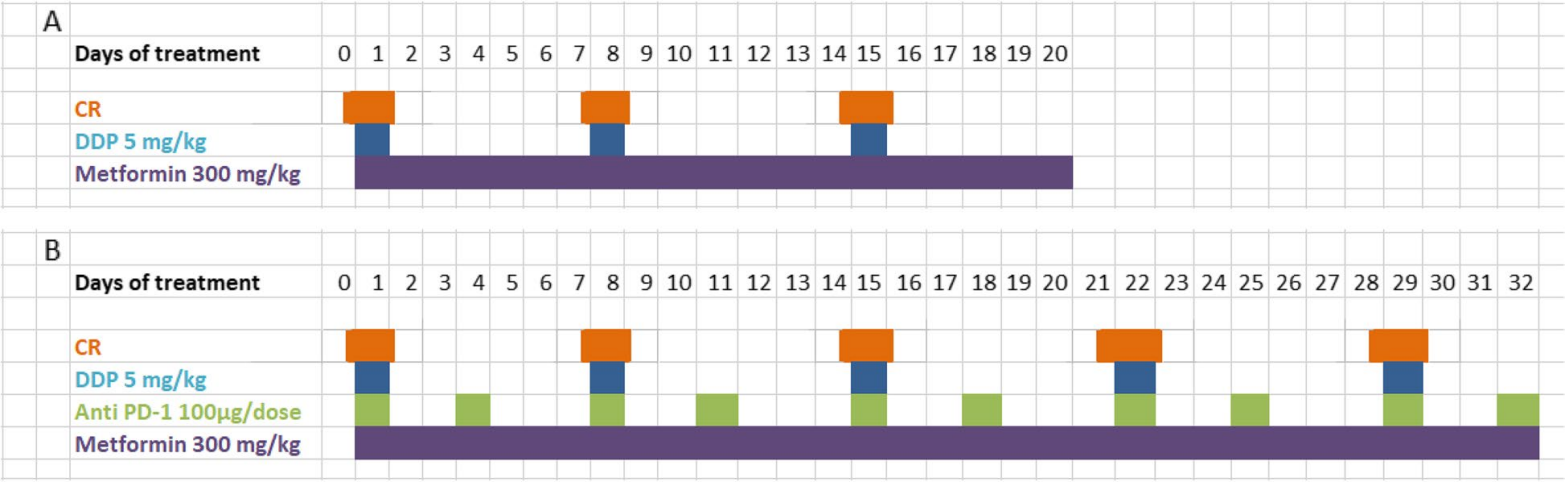
Treatment schedule of in vivo experiments: **A** Mice were treated with chemotherapy (DDP) alone or in combination with metformin and caloric restriction (CR). **B** Mice were treated with chemotherapy (DDP) plus immunotherapy (anti PD-1) alone or in combination with metformin and caloric restriction (CR)

Mice were treated with once a week intraperitoneal (ip) injection of 5 mg/kg cisplatin (CDDP, Sigma- Aldrich), daily with 300 mg/kg of metformin (1,1-Dimethylbiguanide hydrochloride, #D150959 Sigma-Aldrich) by gavage, 100 μg/mouse of anti-mouse PD-1 (CD279, Bio X Cell, Inc.) injected ip twice a week, and 36 h of fasting. Figure 1 reports the treatment schedules.

Tumor diameters were measured with a caliper and volumes were calculated with the following formula for ellipsoid volume: 0.5 × D x d^2^, where D is the long and d is the short diameter.

Tumor volumes were compared at each time point using two-way ANOVA followed by Bonferroni’s a posteriori test.

### Flow cytometry

At sacrifice, the spleens and approximately 150–200 mg of tumor were collected in phosphate buffered saline (PBS). The tumors were cut into small pieces and digested with 250 U/mL collagenase I (C0130 Sigma-Aldrich) at 37°C for 45 min; the digested samples were collected and passed through a 100 μm strainer. The spleens were crushed with the plunger of a 5 mL syringe directly in a 100 μm strainer. For the lysis of red cells, the samples were incubated with 5 mL of Red Blood Cell Lysing Buffer Hybri-Max (Sigma) for 3 min on ice.

The samples were suspended in flow cytometry staining buffer (phosphate-buffered saline with 5% fetal bovine serum) at room temperature (RT) for 10 min. After centrifugation, cells were stained with Zombie Aqua™ Fixable Viability Kit (BioLegend, San Diego, CA, USA, 1:100) at RT in the dark for 30 min. The samples were then washed in flow cytometry staining buffer and resuspended in 100 µL of a cocktail of fluorochrome-conjugate antibodies (Table 1) for 20 min, in the dark at 4°C.

**Table 1:** Fluorochrome-conjugate monoclonal antibodies used for flow cytometry analysis

**Antibody**	**Clonality**	**Clone**	**Company**	**Dilution**
CD45 (FITC)	Monoclonal	30-F11	BioLegend	1:100
CD3 (APC)	Monoclonal	17A02	BioLegend	1:40
CD4 (PerCP/Cyanine5.5)	Monoclonal	RM4-4	BioLegend	1:50
CD8 (PE/Cyanine7)	Monoclonal	YTS156.7.7	BioLegend	1:50
CD27 (Pacific Blue)	Monoclonal	LG.3A10	BioLegend	1:100
CD25 8 APC/Fire 750)	Monoclonal	3C7	BioLegend	1:20
CD11b (Brilliant Violet 650)	Monoclonal	M1/70	BioLegend	1:20
Ly-6G (Brilliant Violet 785)	Monoclonal	1A8	BioLegend	1:150
Ly-6C (Brilliant Violet 570)	Monoclonal	HK1.4	BioLegend	1:20

All antibodies were used as described in *Material and Methods*

The samples were then analyzed with a Cytoflex LX instrument equipped with CytExpert Acquisition software (Beckman Coulter); acquisition was stopped when 50,000 events were collected in the population gate.

**Fig. 2:**
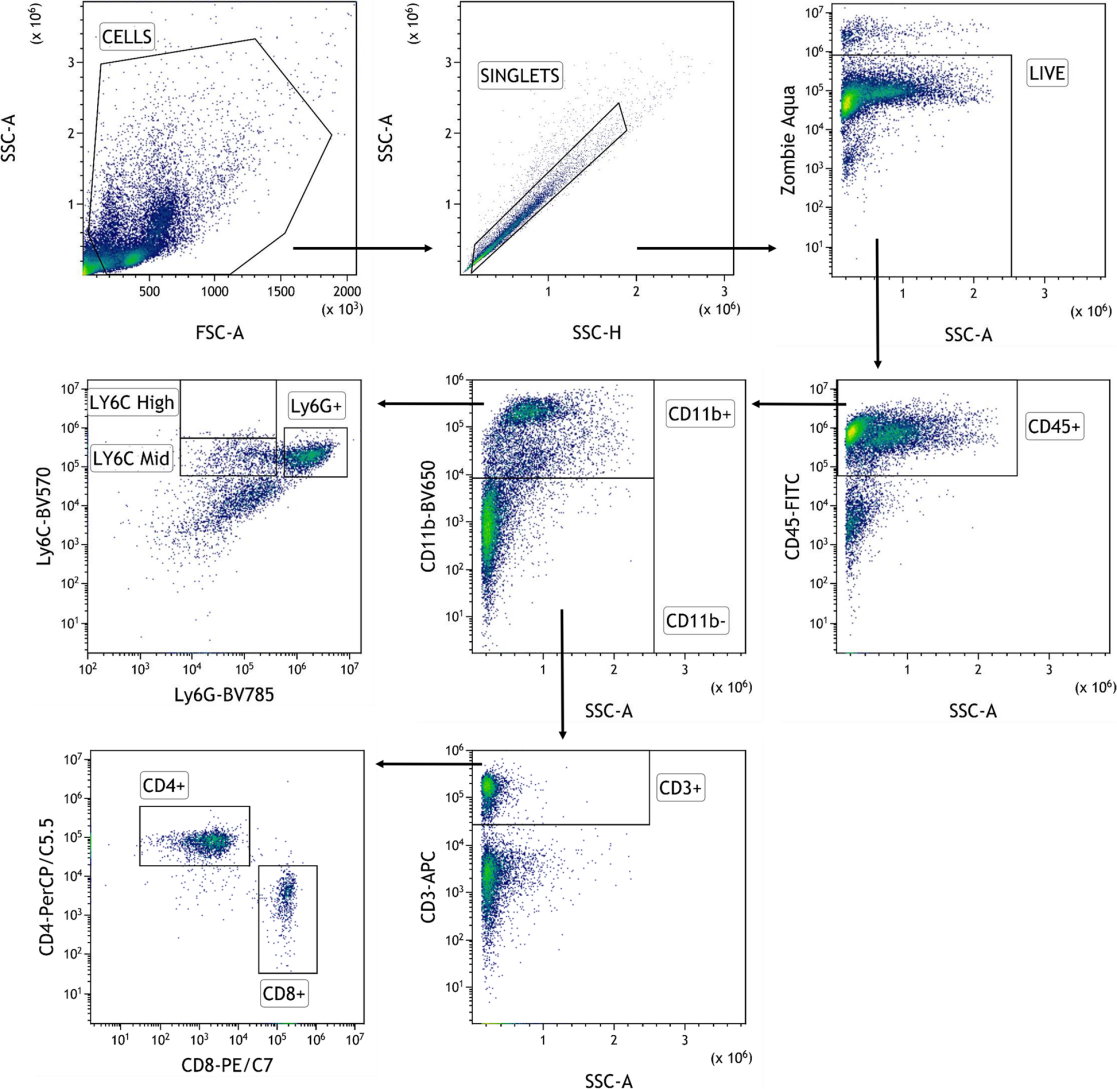
Flow cytometry gating strategy for the identification of immune cell subsets in spleens and tumors: Total cells were first gated on physical parameters, a forward scatter (FSC)/side scatter (SSC) plot, and then gated cells are selected as singlets on the basis of SSC-A and SSC-H. Dead cells are excluded by the Zombie Aqua™ Fixable Viability Kit and leucocytes are identified as CD45^+^. Subset identification is shown for T cells (CD3^+^), CD8^+^ T cells, CD4^+^ T cells, myeloid cells (CD11b^+^), neutrophils (Ly6G^+^), and monocytes (Ly6C.^+^)

Offline analysis was done with FlowJo software version 10 (BD Biosciences). A traditional gating strategy was used to remove aggregates and dead cells, and the percentages of T lymphocytes CD3 + and myeloid cells CD11b + ware evaluated, with the respective cell subpopulations (Fig. 2).

### Immunohistochemical analysis

Tumor samples were fixed in formalin for 24 h, dehydrated and paraffin embedded. Serial sections of 5 µm of thickness were cut and stained with Hematoxylin and Eosin and immunohistochemically with the following primary antibodies: Ki-67 (Thermoscientific, clone sp6, rabbit monoclonal, #RM-9106-S, 1:500), Cleaved Caspase-3 (Cell Signaling, clone Asp175, rabbit polyclonal, #9661, 1:2000) Glutathione Peroxidase 4 (Abcam, rabbit monoclonal, ab125066, 1:2250), and γH2AX (Cell Signaling, Ser139 clone 20E3, rabbit monoclonal, #9718, 1:500). Sections underwent deparaffinization and heat-induced epitope retrieval for 40 min at 96°C (Dewax and HIER Buffer H, Thermo Scientific, Runcorn, UK, cat. no. TA-999-DHBH). Endogenous peroxidase activity was blocked by incubating sections in 3% H2O2 for 10 min. Slides were rinsed, incubated with phosphate-buffered saline (PBS) containing 10% normal serum for 30 min at room temperature to reduce nonspecific background staining, and then incubated for 90 min at room temperature with the primary antibodies. Sections were then incubated with a biotinylated secondary antibody, labeled by the avidin–biotin–peroxidase procedure (VECTASTAIN Elite ABC-Peroxidase Kit Standard, Vector Laboratories, cat. no. VC-PK-6100-KI01). The immunoreaction was visualized with 3,30-diaminobenzidine (DAB, Peroxidase DAB Substrate Kit, Vector Laboratories, cat. no. VC-SK-4100-KI01) substrate, and sections were counterstained with hematoxylin. Known positive control sections were included in each immunolabeling assay. The percentage of positive/negative cells was evaluated at the light microscopy considering three 200 × microscopic fields randomly selected within the tumor and avoiding necrotic areas. The evaluation was made in a blind fashion, without information about the treatment groups.

### Statistical analysis

Statistical analyses were done with GraphPad Prism 7. Figure legends specify which test was used for each experiment. Differences between groups were considered statistically significant when p < 0.05.

## Results

### In vitro effects of metformin and caloric restriction on cell viability, metabolism and on the activation of pro-tumor pathways

We initially examined whether metformin and/or caloric restriction (CR) had distinct effects in cells with different *LKB1* mutational status. We used two cell lines obtained in our laboratory from lung nodules of transgenic mice, characterized by *KRAS*^G12D mut^ (K) and *KRAS*^G12D mut^/*LKB1*^del^ (KL) genetic background [34]. To determine the effect on cell viability, both cell lines were treated with 2 mM of metformin and a 50% reduction of glucose, glutamine and FBS in the cell media for 72 h.

**Fig. 3:**
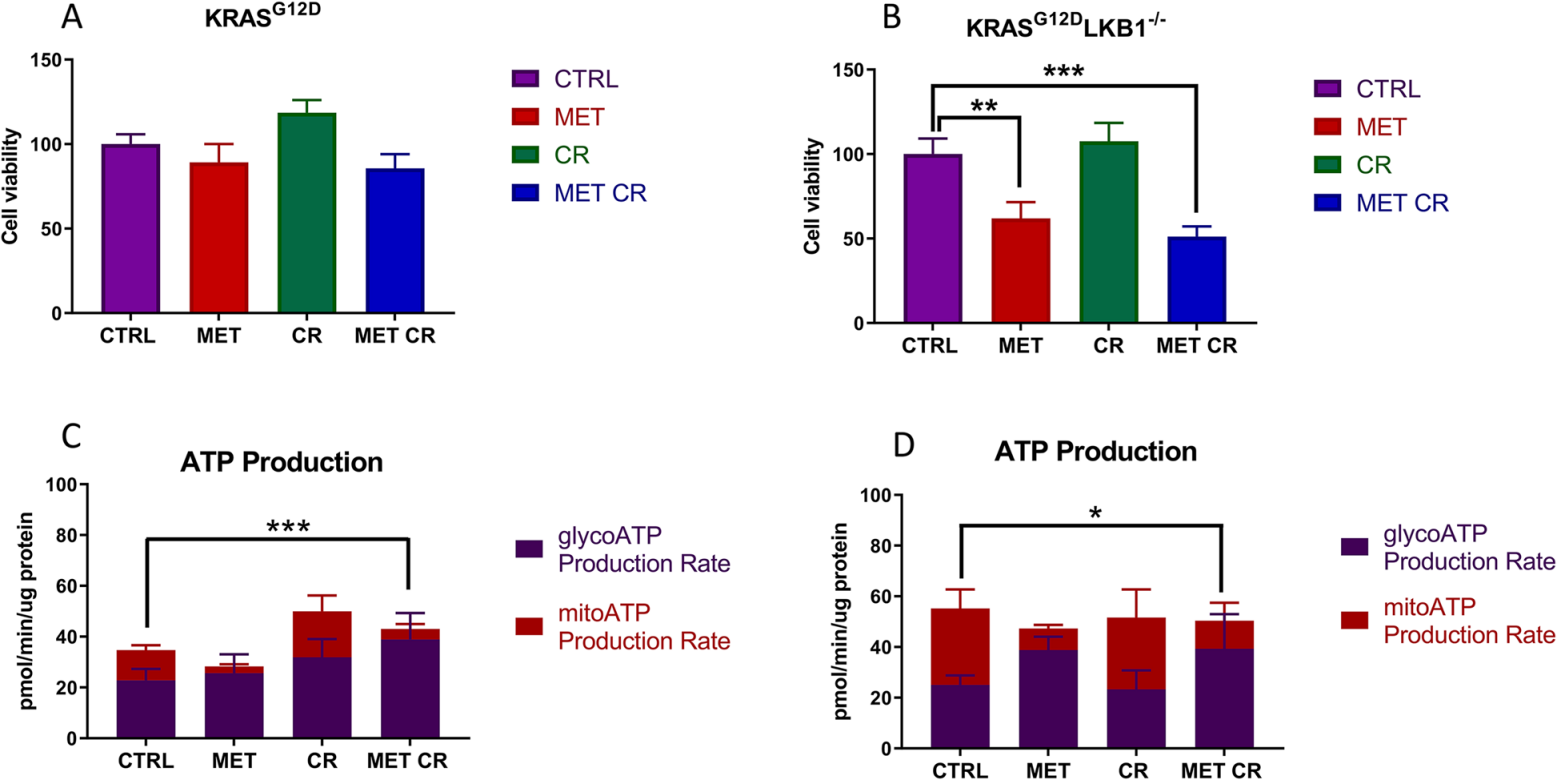
In vitro effect of metformin and CR treatments on cell viability and metabolism: Cell viability measured by MTS assay after 72 h of treatment with 2 mM of metformin (MET) and 50% of caloric restriction (CR) in KRAS^G12D^ (**A**) and KRAS^G12D^/LKB1^del^ (**B**) cell lines. One-way ANOVA was used for statistical analysis (**p < 0.01, *** *p* < 0.001). Error bars indicate SD. Seahorse XF Real-Time Assay was done after 24 h of treatment with 2 mM of metformin (MET) and 50% caloric restriction (CR) in KRAS^G12D^ (**C**) and KRAS^G12D^/LKB1^del^ (**D**) cell lines. Two-way ANOVA was used for statistical analysis (* *p* < 0.05, **p < 0.01, *** *p* < 0.001). Error bars indicate SD

As shown in Fig. 3A, B metformin induced cell death only in the KL cell line, and the combination with CR further increased this effect; the same conditions did not affect K cell line growth.

To investigate the metabolic differences characterizing K and KL cells, we measured the total ATP production rate distinguishing between the fraction of ATP produced from mitochondrial oxidative phosphorylation (OXPHOS) and glycolysis. At basal level, K and KL cell lines had similar glycoATP production rates, while KL cells had higher mitoATP production. As expected, 24 h of treatment with metformin induced a reduction of ATP obtained through OXPHOS in both cell lines, while CR did not affect ATP production, independently of *LKB1* status. The combination of metformin and CR resulted in similar energetic responses in K and KL cells, with a rise in the glycoATP production rate (Fig. 3C, D).

**Fig. 4:**
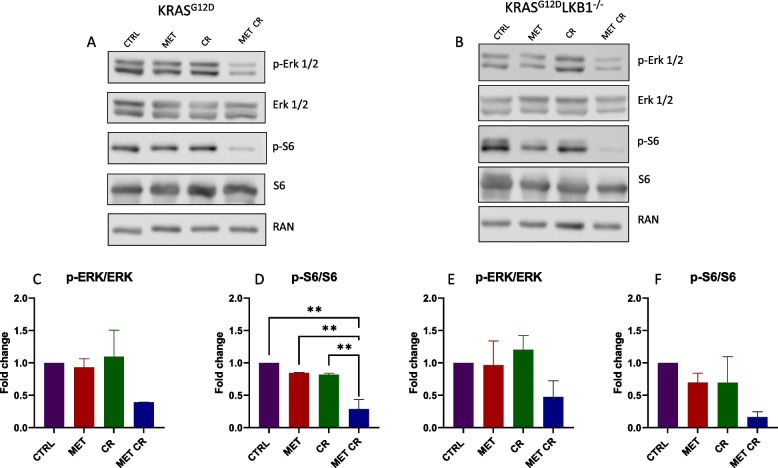
In vitro effect of metformin and CR treatments on mTOR and MAPKs proliferative pathways: Representative immunoblot analysis of total and phosphorylated forms of extra-cellular signal-regulated kinase (ERK 1/2) and S6 ribosomal protein (S6) in KRAS^G12D^ (**A**) and KRAS^G12D^/LKB1^del^(**B**) cell lines. **C**-**F** Bar graphs of the quantification analysis of the active forms of ERK and S6 normalized on RAN, used as housekeeping. Proteins were extracted after 72 h of treatment with 2 mM of metformin (MET) and 50% caloric restriction (CR). Non-treated cells were used as control. One-way ANOVA was used for statistical analysis (**p < 0.01). Error bars indicate SD

The main antiproliferative mechanism of metformin is mediated by the activation of AMPK and the resulting inhibition of mTOR; therefore, we measured the levels of phospho-S6 (p-S6) downstream of mTOR. P-S6 was reduced after metformin treatment only in KL cells, while the combination of metformin and CR induced reductions in both cell lines, with a greater effect was in the absence of LKB1. In addition, to verify the possible effects of metformin and CR on the MAPKs pathway, highly activated in the presence of constitutive activation of KRAS, we measured phospo-ERK1/2 (p-ERK1/2). Immunoblotting analysis indicated that either metformin or CR alone did not affect p-ERK, while the combination resulted in its reduction, and the effect was independent of *LKB1*-status (Fig. 4).

**Fig. 5:**
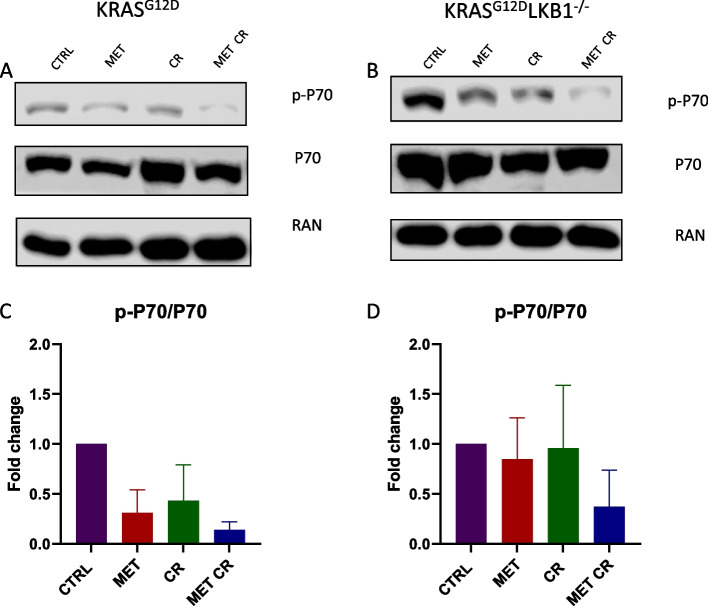
In vitro effect of metformin and CR treatments on mTOR proliferative pathway: Representative immunoblot analysis of total and phosphorylated forms of P70 S6 kinase (P70) in KRAS^G12D^ (**A**) and KRAS^G12D^/LKB1^del^(**B**) cell lines. **C**, **D** Bar graphs of the quantification analysis of the active forms of P70 normalized on RAN, used as housekeeping. Proteins were extracted after 72 h of treatment with 2 mM of metformin (MET) and 50% caloric restriction (CR). Non-treated cells were used as control. One-way ANOVA was used for statistical analysis and no statistically significant results were highlighted. Error bars indicate SD

**Fig. 6:**
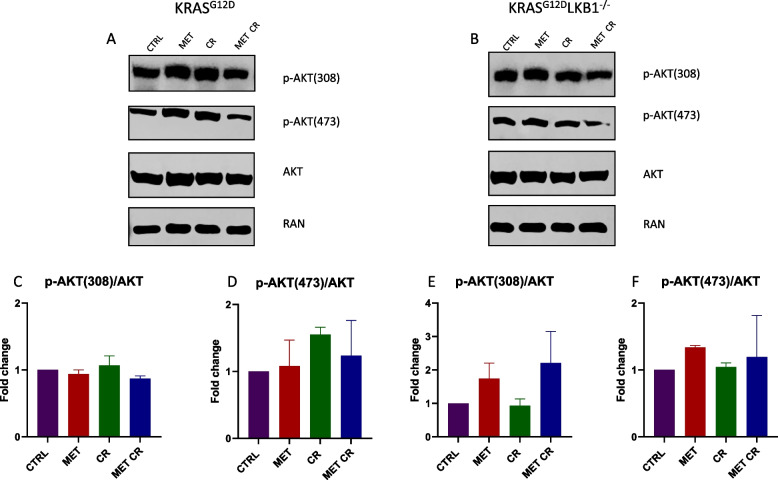
In vitro effect of metformin and CR treatments on PI3K/AKT proliferative pathway: Representative immunoblot analysis of total and different phosphorylated forms of the serine/threonine kinase AKT in KRAS^G12D^ (**A**) and KRAS^G12D^/LKB1^del^(**B**) cell lines. **C**-**F** Bar graphs of the quantification analysis of the different active forms of AKT normalized on RAN, used as housekeeping. Proteins were extracted after 72 h of treatment with 2 mM of metformin (MET) and 50% caloric restriction (CR). Non-treated cells were used as control. One-way ANOVA was used for statistical analysis and no statistically significant results were highlighted. Error bars indicate SD

To further investigate the involvement of the pathway of mTOR in the response to metformin alone or in combination with CR, we assessed the activation of the serin/threonine kinase AKT. AKT is downstream of PI3K, and when activated, it can subsequently trigger mTOR. Additionally, we evaluated the activation of P70, downstream of mTOR. Also in this case, the modulation of the proteins considered appeared to be independent on LKB1 status. In fact, the treatment with 2mM of metformin or 50% CR induced a reduction in P70 activation in both cell lines. The combination of metformin and CR resulted in a further decrease (Fig. 5). The activation of AKT did not appear to be modulated in the presence of both metformin and/or CR (Fig. 6).

### Effect of metformin and caloric restriction on cisplatin response

To assess the in vivo effect of the combination of metformin and CR on cisplatin (DDP) response, we injected immunocompetent mice subcutaneously with K or KL cells. The animals were randomized into eight treatment groups: control, DDP, metformin, CR, metformin/CR DDP/metformin, DDP/CR and DDP/metformin/CR. The treatment schedule consisted in metformin 300 mg/Kg daily and DDP 5 mg/Kg once a week during the CR, for three weeks. Since it was not possible to limit the caloric intake of each individual mouse, 36 h fasting was used to reduce the systemic availability of nutrients.

**Fig. 7:**
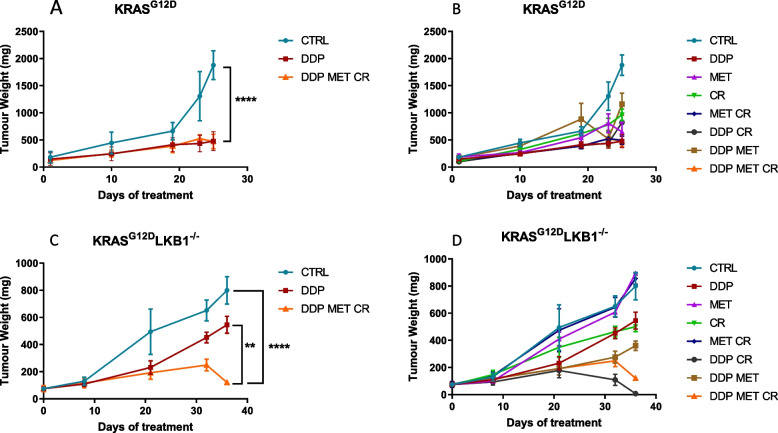
Effect of metformin and CR plus chemotherapy in subcutaneous K and KL tumors: Tumor growth inhibition in KRAS^G12D^ (**A**, **B**) and KRAS^G12D^/LKB1^del^ (**C, D**) subcutaneously injected in immunocompetent mice (*N* = 9). For three weeks metformin (MET) was administered daily at 300 mg/kg, cisplatin (DDP) 5 mg/kg once a week and caloric restriction (CR) consisted of 36 h of fasting, once a week. Two-way ANOVA was used for statistical analysis (**p < 0.01, ****p < 0.0001). Error bars indicate SEM. The most statistically significant results have been reported in panels A and C. Single treatments, reported in panels B and D, did not induce a statistically significant reduction in tumor growth when compared to control or DDP groups

As expected, K tumors were more sensitive to DDP than KL ones, but the co-treatment with metformin and CR resulted in a significant increase in DDP response only in *LKB1-*deleted tumors (Fig. 7).

**Fig. 8:**
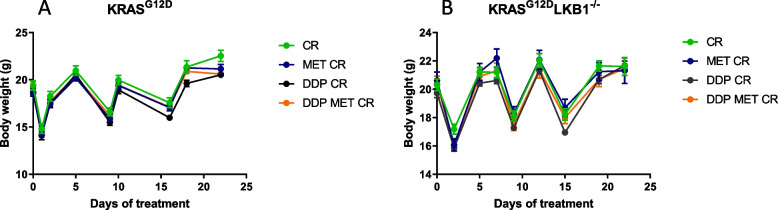
Graph of body weight of mice bearing K and KL tumors: Body weight of KRAS^G12D^ (**A**) and KRAS^G12D^/LKB1^del^ (**B**) mice used in Fig. 7 submitted to CR alone or in combination with DDP or/and metformin. The treatment schedule was well-tolerated, as the body weight lost during the fasting period was regain within 24 h

Treatment tolerability was evaluated through body weight measurements; as expected, fasting caused a considerable weight loss (3–4 g), which, however was recovered in 24 h after food reintroduction (Fig. 8). Fasting lowered the glucose levels in blood by roughly 50% and they returned to normal in the 24 h after fasting (data not shown).

**Fig. 9:**
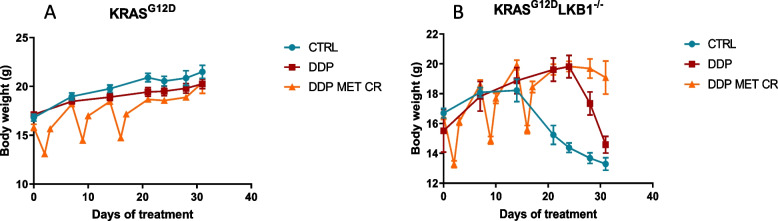
Graph of body weight of mice bearing K and KL tumours: Body weight of KRAS^G12D^ (**A**) and KRAS^G12D^/LKB1^del^ (**B**) mice used in Fig. 10. Untreated mice (CTRL) were compared to mice treated with DDP or DDP/MET/CR. The treatment schedule was well-tolerated, as the body weight lost during the fasting period was regain within 24 h. Intramuscular inoculation of KL cells, induced a massive weight loss in immunocompetent mice

**Fig. 10:**
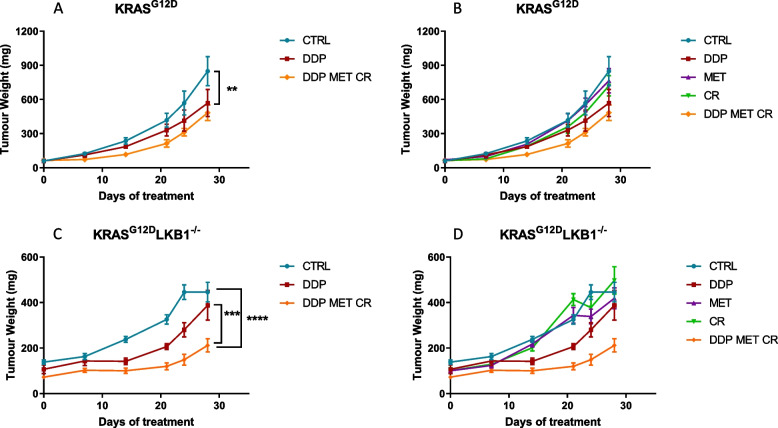
Effect of metformin and CR plus chemotherapy in intramuscular K and KL tumors: Tumor growth inhibition in KRAS^G12D^ (**A**, **B**) and KRAS^G12D^/LKB1^del^ (**C**, **D**) intramuscularly injected in immunocompetent mice (*N* = 9). For three weeks metformin (MET) was administered daily at 300 mg/kg, cisplatin (DDP) 5 mg/kg once a week and caloric restriction (CR), consisted of 36 h of fasting, once a week. Two-way ANOVA was used for statistical analysis (**p < 0.01, *** *p* < 0.001, ****p < 0.0001). Error bars indicate SEM. The most statistically significant results have been reported in panels A and C. Single treatments, reported in panels B and D, did not induce a statistically significant reduction in tumor growth when compared to control or DDP groups

We repeated the experiment changing the side of cell injection and inoculating the same cell lines intramuscularly to avoid tumor ulcers formed with the sc implanted cells. Given the results obtained with subcutaneously growing tumors, the mice were distributed in five groups, concentrating the efforts on the combination of metformin and CR: controls, DDP, metformin, CR and the combination of DDP/metformin/CR. Confirming the previous data, treatments were well tolerated and the body weight loss during the fasting were regained after food reintroduction (Fig. 9). Importantly, K tumors confirmed to be more sensitive to DDP than KL ones, while the addition of metformin and CR enhanced the effect of chemotherapy only in *LKB1*-deleted tumors. In KL tumours the beneficial effect of the combination lasted one week after the end of the treatment. In fact, while the end of the treatment with DDP induce a rapid resumption of tumour growth, tumours treated with the combination exhibited a more controlled growth after the end of the treatment (Fig. 10).

**Fig. 11:**
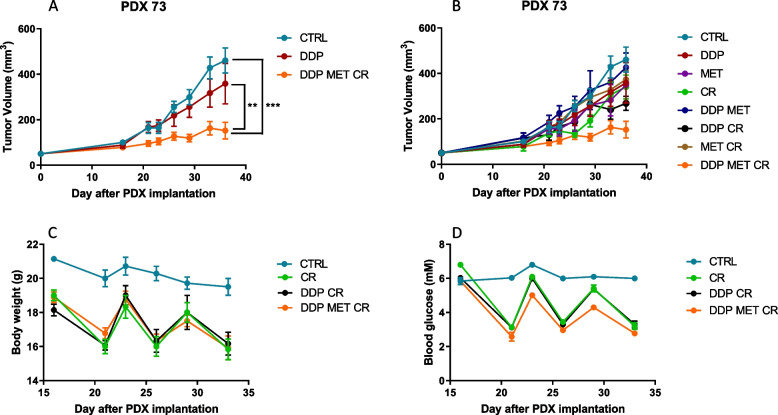
Effect of metformin and CR plus chemotherapy in subcutaneous KL PDX tumor: **A**, **B** Tumor growth inhibition in KL PDX implanted subcutaneously in immunocompromised mice. For three weeks metformin (MET) was administered daily at 300 mg/kg, cisplatin (DDP) 5 mg/kg once a week and caloric restriction (CR), consisted of 24 h of fasting, once a week. Caloric restriction alone or plus metformin and chemotherapy reduced (**C**) body weight and (**D**) blood glucose in immunecompromised mice. Two-way ANOVA was used for statistical analysis (**p < 0.01, *** *p* < 0.001). Error bars indicate SEM. The most statistically significant results have been reported in panel **A**. Single treatments, reported in panels **B**, did not induce a statistically significant reduction in tumor growth when compared to control or DDP groups

We then investigated the effects of metformin and CR in patient-derived xenografts (PDX) *LKB1* mutated (PDX73), a model that closely mirrors the structure and heterogeneity of human tumors. We followed the same treatment schedule as in the previous in vivo experiments. CR combined with metformin and DDP had the highest effect on PDX73 growth (Fig. 11A, B). Metformin and fasting reduced blood glucose levels and mouse body weight. Blood glucose reduction induced by fasting reversed within 24 h, whereas body weight was not completely regained, following the pattern of progressive body weight loss in control mice (Fig. 11C, D).

### Effects of metformin and caloric restriction on chemo-immunotherapy response

Since chemo-immunotherapy is now widely used in daily clinical practice in NSCLC patients with PD-L1 expression lower than 50%, we examined if the combination of metformin and CR improved the effect of chemo plus immunotherapy in *LKB1*-deleted tumors. As in the previous experiments, CR consisted of 36 h of fasting, metformin was given daily at 300 mg/kg and DDP at 5 mg/kg once a week during the CR. Anti PD-1 was administrated twice a week at 100 μg/mouse; we prolonged the schedule, treating the mice for five weeks. The mice were inoculated intramuscularly with K or KL tumors and divided into five groups: control, DDP/anti PD-1, DDP/anti PD-1/metformin, DDP/anti PD-1/CR and DDP/anti PD-1/metformin/CR.

**Fig. 12:**
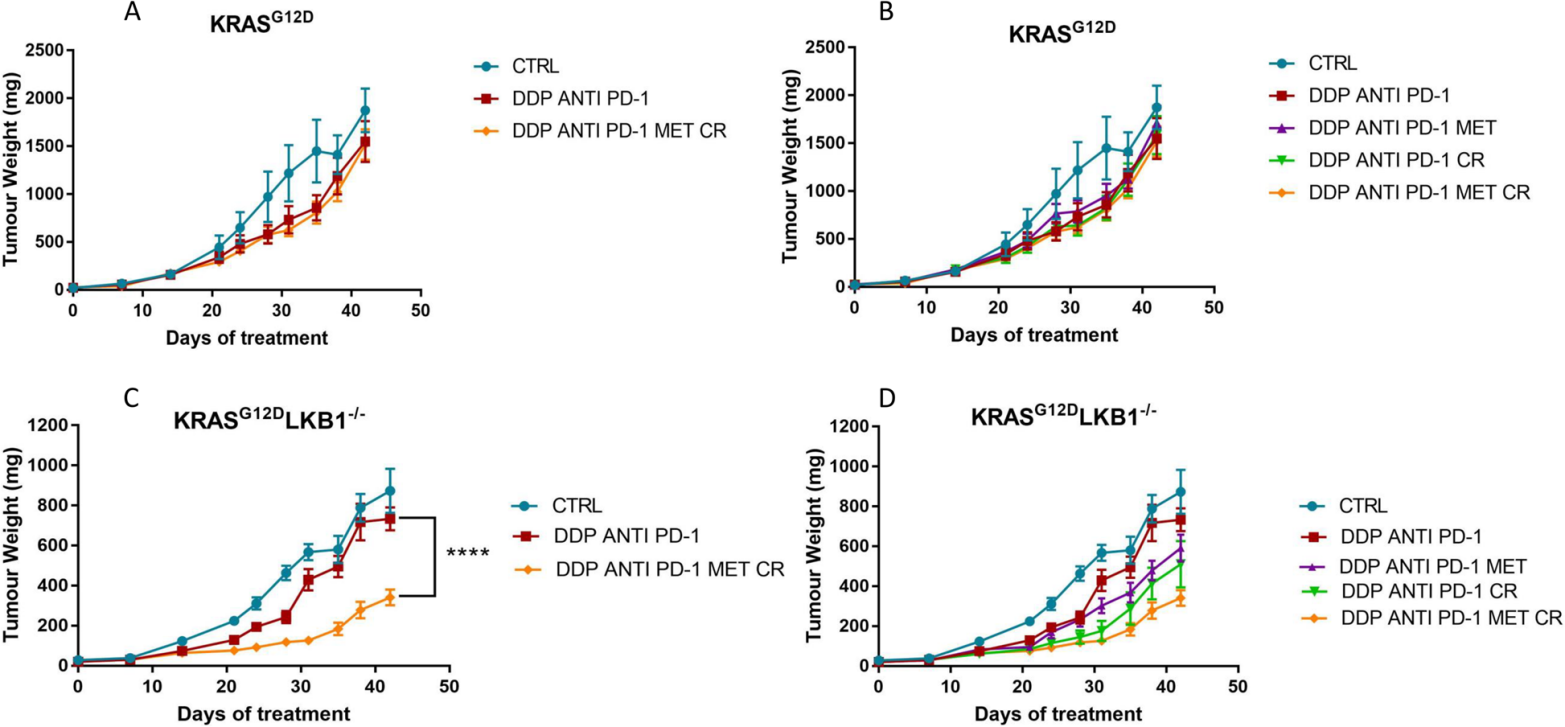
Effect of metformin and CR plus chemo-immunotherapy in intramuscular K and KL tumors: Tumor growth inhibition in KRAS^G12D^ (**A**, **B**) and KRAS^G12D^/LKB1^del^ (**C**, **D**) intramuscularly injected in immunocompetent mice (*N* = 10). For five weeks metformin (MET) was administered daily at 300 mg/kg, cisplatin (DDP) 5 mg/kg once a week, anti PD-1 at 100 μg/dose twice a week, while the caloric restriction (CR) consisted of 36 h of fasting once a week. Two-way ANOVA was used for statistical analysis (**p < 0.01, ****p < 0.0001). Error bars indicate SEM. **A**, **B** Non statistically significant effects were observed in K tumours. For KL tumours, the most statistically significant results have been reported in panels C. **D** CR increases the effect of DDP/anti PD-1 combination (DDP/anti PD-1 vs DDP/anti PD-1/MET **p < 0.01) but (**C**, **D**) the best effect were reached with the DDP/anti PD-1/MET/CR combination

As shown in Fig. 12, while the combination of DDP/anti PD-1 only slightly impaired the growth of both K and KL tumors, the addition of metformin and CR significantly increased the response only in *LKB1*-mutated tumors.

**Fig. 13:**
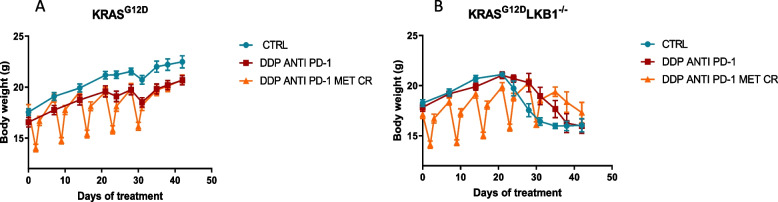
Graph of body weight of mice bearing K and KL tumors: Body weight of KRAS^G12D^ (**A**) and KRAS^G12D^/LKB1^del^ (**B**) mice used in Fig. 12. No-treated mice (CTRL) were compared to mice treated with DDP/anti PD-1 or DDP/anti PD-1/MET/CR. The treatment schedule was well-tolerated, as the body weight lost during the fasting period was regain within 24 h. Intramuscular inoculation of KL cells, induced a massive weight loss in immunocompetent mice

In this case too, all treatments were well tolerated and weight lost during fasting was regained in 24 h after food reintroduction (Fig. 13).

**Fig. 14:**
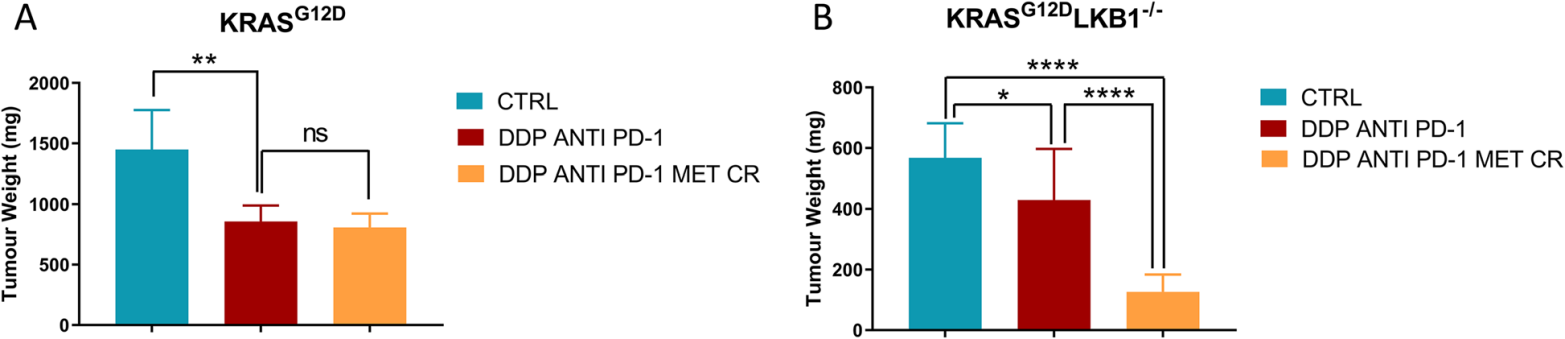
Best treatment effect obtained in intramuscular K and KL tumours in immunocompetent mice: KRAS^G12D^ (**A**) and KRAS^G12D^/LKB1^del^ (**B**) tumor volumes on days 35 and 31 of treatment respectively, the time-point when treatment benefit was greatest. Two-way ANOVA was used for statistical analysis (*p < 0.05, **p < 0.01, **** *p* < 0.0001). Error bars indicate SEM

In K tumors the best effect of DDP/anti PD-1 was achieved on day 35th of treatment but, as underlined before, the addition of metformin and CR did not reduce the tumor growth compared to chemo-immunotherapy (Fig. 14A). The best treatment effect of the combination in KL tumors was reached at the 31st day of treatment (Fig. 14B) but, notably the benefit of the addition of metformin and CR combination lasted beyond the end of treatment (Fig. 12B-D).

To try to underline the reason for the increased sensitivity of LKB1-deleted tumor to the addition of metformin and CR, we analyzed the morphology by histology and the levels of different markers by immunohistochemistry. While we did not observe significant differences between K and KL tumors in terms of percentage of necrosis, expression of activated caspases (as indicator of apoptosis) and gH_2_Ax (as indicator of DNA damage induction), we found a significant reduction in the expression of GPX4 (a protein known to be involved in cellular protection against oxidative stress) in KL versus K tumors.

**Fig. 15:**
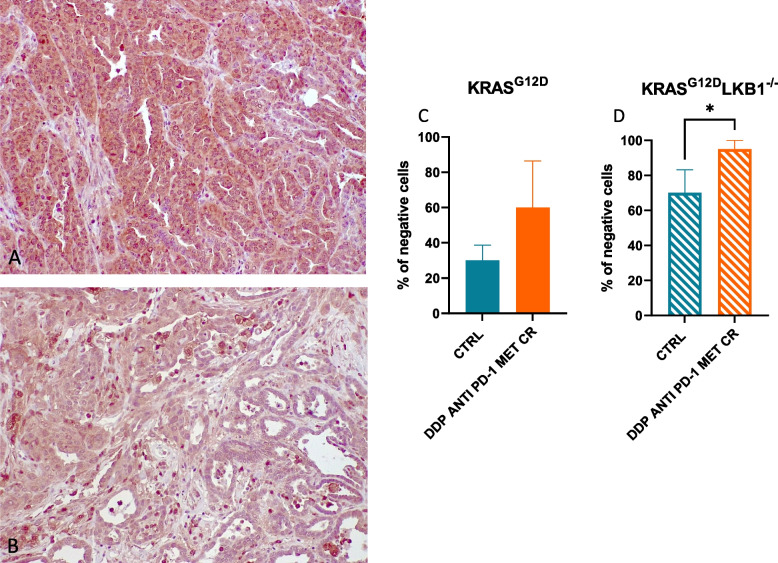
Quantification of GPX4 expression in K and KL tumors: Representative image of immunohistochemical staining for GPX4 of K (**A**) and KL (**B**) tumors. **C**, **D** The percentage of negative cells are represented as the mean of three different samples. Unpaired t test was used for statistical analysis (* *p* < 0.05). Error bars indicate SD

In fact, at the baseline, KL tumors exhibited a lower amount of GPX4 compared to K tumors and, the treatment with metformin and caloric restriction (CR) led to a further decrease in GPX4 levels that was more pronounced in tumor lacking LKB1 (Fig. 15).

### Characterization of the immune component

Different studies have demonstrated a correlation between loss of *LKB1* and a particular immune TME, with tumors lacking LKB1 defined as “cold”. To confirm whether this particular TME also characterized our model, we have evaluated the percentage of total immune cells (CD45^+^), in which we distinguished the percentage of myeloid (CD11b^+^) and non-myeloid (CD11b^−^) components. In addition, at the level of the myeloid population, we specifically evaluated the percentages of neutrophils (CD11b^+^ Ly6G^+^) and monocytes (CD11b^+^ Ly6C^+^), while at the level of the non-myeloid cells, which are mainly lymphoid populations, we focused on T-helper (CD3^+^ CD4^+^) and T-cytotoxic (CD3^+^ CD8^+^) subsets.

**Fig. 16:**
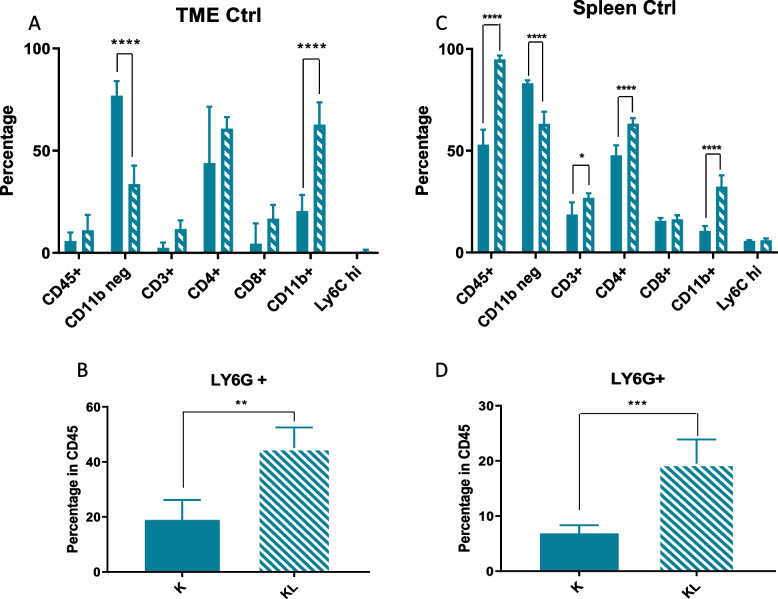
TME and spleen immune characterization of K and KL untreated mice: **A**, **B** TME immune profile of KRAS^G12D^ and KRAS^G12D^/LKB1^del^ tumours injected intramuscularly; **C**, **D** spleen immune profile of immunocompetent mice bearing KRAS^G12D^ and KRAS^G12D^/LKB1^del^ tumours; samples from the experiment in Fig. 12 were collected and processed for FACS analysis of lymphoid and myeloid infiltrating immune cells (see Fig. 2 for the gate strategy). One-way and two-way ANOVA were used for statistical analysis (**p < 0.01, ****p < 0.0001). Error bars indicate SD

Comparing K and KL tumors, immune cell flow cytometry analysis confirmed how, despite a similar percentage of CD45^+^ cells, KL tumors had a colder TME given by a smaller percentage of CD11b^−^ cells and a larger percentage of CD11b^+^ cells, related mainly to an increase of neutrophils (Fig. 16A, B).

As the spleen is a major source and reservoir of circulating and infiltrating immune cells [38], we investigated the immune composition of this organ in our models. There was an increase of leucocytes in mice bearing KL tumors, and as in the TME, the spleens of these mice had higher levels of CD11b^+^ cells, related to a larger number of neutrophils, and lower levels of CD11b^−^ cells which however, were not related to an increase of CD3^+^ cells (Fig. 16C, D).

**Fig. 17:**
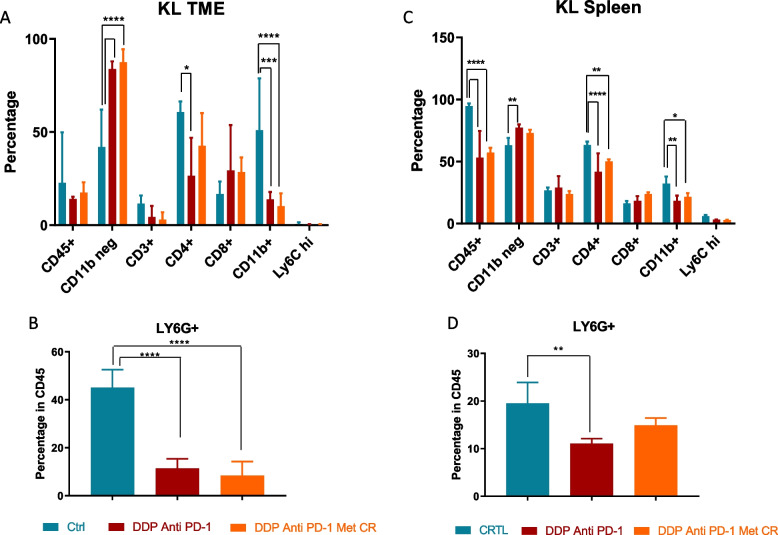
TME and spleen immune characterization of KL treated mice: **A**, **B** TME and (**C**, **D**) spleen immune modulation in mice bearing KL tumors; samples from the experiment in Fig. 12 were collected and processed for FACS analysis of lymphoid and myeloid infiltrating immune cells (Fig. 2 for the gate strategy). One way and two-way ANOVA were used for statistical analysis (*p < 0.05, *** *p* < 0.001 ****p < 0.0001). Error bars indicate SD

As pointed out previously, different studies have highlighted the immune modulation effects of metformin and caloric restriction. Our results illustrate how the two metabolic stressors only minimally act on the KL tumor microenvironment. The treatment with DDP/anti PD-1 led to significant modulation of the immune infiltrating cells, with an increase of CD11b^−^ cells and a decrease of CD11b^+^ ones. In the myeloid component there was a reduction of neutrophils, while the increase of the non-myeloid population (CD11b^−^) was not related to the recruitment of CD3^+^/CD8^+^ or CD3^+^/CD4^+^ cells (Fig. 17A, B).

The significant immune modulation in the TME with DDP/anti PD-1 co-treatment was only slightly improved by the addition of metformin and CR, with a further reduction of the neutrophils (CD11b^+^ Ly6G^+^) (Fig. 17B).

In the spleen DDP/anti PD-1 led to a decrease of total immune cells (CD45^+^) and, like in the TME, there was an increase in the percentage of CD11b^−^ cells and a reduction of CD11b^+^, this last related with a decrease of CD11b^+^ Ly6G^+^ cells (Fig, 17C, D).

## Discussion

The introduction of immunotherapy and the development of targeted therapies have improved the treatment of NSCLCs. However, new therapeutic strategies for the *LKB1*^mut^ subgroup of patients are still a medical need, the latter being much less responsive even to these new treatments. Given the key role of LKB1 in the regulation of cell metabolism, we investigated in *KRAS*^mut^/*LKB1*^mut^ NSCLC (KL) preclinical models whether the introduction of metabolic stressors like metformin and caloric restriction, in combination with current therapies, could lead to a better tumor response.

Previous data from our group showed that metformin not only enhanced the effect of cisplatin but also counteracted the onset of the resistance in NSCLC *KRAS*^mut^/*LKB1*^del^ cells [20].

Focusing on the in vitro effect of the metabolic stressors, our data show that CR alone is not able to impair cell growth in KL cells, whereas metformin alone induced cell death and the addition of CR amplified its effect. In contrast, cells with a wild-type *LKB1* status were not sensitive to either metformin or CR, or even to the combination.

Our results obtained with the Real-Time ATP Rate SeaHorse Assay led us to speculate that KL cells depend more on ATP produced from OXPHOS. In fact, compared to K cells they have a more active metabolism, with higher mitoATP production. The reduction of the ATP from mitochondria, with metformin or metformin and CR, induced an increase of glycolysis that seemed, however, not sufficient to satisfy the cell’s metabolic demand, causing a metabolic crisis that leads to cell death after 72 h of treatment.

At the molecular level, the combination of metformin and CR reduced the activation of both the MAPKs and mTOR pathways independently of the status of *LKB1*, indicating that the effect on cell viability in KL cells is related not only to the action on these two proliferative pathways but is the result of the modulation of other key LKB1-dependent cellular mechanisms, still under investigation.

Using both syngeneic and PDX models, we demonstrate that the addition of metformin and CR strongly improves the response to chemotherapy in *LKB1* impaired tumors, while the same combination has no enhancing effect in tumors with wild-type *LKB1* status. Of note, in a previous study we showed how metformin enhanced DDP’s effect on the same KL PDX, but only after several treatment cycles and with a higher dosage. Here we highlight the synergistic role of metformin and CR in increasing DDP’s effects on KL NSCLC models.

The combination of metformin and CR was also effective with chemo-immunotherapy in the KL syngeneic model, a combination that has not been tested in the PDX model, as human tumors have the limitation of needing immunocompromised mice to grow.

A previous study conducted in our laboratory highlighted how metformin could induce apoptosis in the absence of LKB1 [20]. However, we cannot exclude the involvement of different mechanisms of cell death after the treatment with the combination, in our KL in vivo model. Various studies have linked the absence of LKB1 to a higher sensitivity to oxidative stress [39, 40] and, additionally, a recent study by J. W. Kim et al. proposed the possibility of exploiting ferroptosis as a valid way to induce cell death in NSCLC through the inhibition of GPX4 [41]. Consistent with a higher sensitivity to oxidative stress, we observed a lower amount of GPX4 in KL tumors at basal levels compared to K ones. Considering that we observed a further reduction of GPX4 in KL tumors after treatment with metformin and CR in combination with chemo-immunotherapy, we can hypothesize the involvement of ferroptosis as one of the cell death mechanisms implicated in the inhibition of tumor growth after the combination treatment. Further data are needed to fully support this hypothesis.

As reported in the literature, our analysis of the tumor immune infiltration confirms that the absence of LKB1 characterizes a particularly “cold” TME. In fact, compared to *KRAS*^G12D^/*LKB1*^wt^ (K) tumors in KL ones for the same number of infiltrating immune cells, we found a greater presence of the immunosuppressive CD11b^+^ cells related to a larger number of neutrophils, and fewer CD11b^−^ cells, although not related to the subpopulation of T cells that we considered. Analysis of the spleen, as the main supplier of circulating and infiltrating immune cells, showed an immune profile comparable to that found in the tumor microenvironment. In fact, despite a larger number of CD45^+^ cells, the spleens of KL bearing mice have been found to be richer in CD11b^+^ and poorer in CD11b^−^ cells.

While chemo-immunotherapy can increase the non-myeloid (CD11b^−^ cells) and reduce neutrophils (CD11b^+^ Ly6G^+^ cells) in the KL TME, the addition of metformin and CR did not boost this effect more than minimally.

We have preliminary data showing that the addition of metabolic stressors to immunotherapy alone does not have a durable enhancing effect in tumors lacking LKB1 (data not shown). This suggests that in the absence of LKB1 the immune-modulation effects of metformin and CR are not enough to induce the switch from a “cold” to an “hot” TME, a mechanism that is considered the leading cause of improvement in the immunotherapy response.

Moreover, the beneficial effect of metformin and CR plus chemotherapy in the KL PDX model further underlines how the greater KL tumor response to the combination is not linked to the modulation of T cells in the KL TME, but is more likely to be related to the inability of these tumors to overcome metabolic stress.

Very recent publications stress a link between *LKB1* mutations and the onset of cachexia, characterized by adipose and lean mass loss [42, 43]. Although beyond the scope of the present work, in agreement with this new finding, we did find that the inoculation of our KL cells induced a massive body weight loss in immunocompetent mice when tumors reached more than 400 mg. The addition of metformin and CR combined with chemo alone or chemo-immunotherapy delays the onset of this phenomenon, a benefit that might be related to a smaller tumor mass in the treated mice. Elucidating the mechanism of the association between the absence of LKB1 and the onset of cachexia could be extremely important to preserve the general well-being of patients, as well as finding new therapeutic strategies to counteract the cancer. This aspect is currently being actively investigated.

## Conclusion

In all, our findings show how metformin and CR could be taken into consideration to improve the therapeutic response of a particular subgroup of NSCLC patients who lack LKB1, exploiting the inability of these tumors to overcome metabolic stress.

Metformin has been associated to an improvement of the clinical outcome in cancer patients treated with immune checkpoint inhibitors [44, 45], currently, we are conducting a clinical study in NSCLC patients impaired in LKB1, to assess the safety and efficacy of a low-calory diet or metformin, or both, in addition to chemo-immunotherapy [46].

## Data Availability

Data generated or analyzed during this study are included in this published article and its supplementary information files. All the raw data are available upon request from the corresponding author.

## Abbreviations

NSCLC: Non-small cell lung cancer
KRAS: Kirsten rat sarcoma virus
STK11: Serine/threonine kinase 11
LKB1: Liver kinase B1
TME: Tumor microenvironment
CR: Caloric restriction
AMP: Adenosine Monophosphate
ATP: Adenosine triphosphate
FMD: Fasting-mimicking diet
FBS: Fetal Bovine Serum
MTS: 3-(4,5-Dimethylthiazol-2-yl)-5-(3-carboxymethoxyphenyl)-2-(4-sulfophenyl)-2H-tetrazolium
SD: Standard deviation
SEM: Standard error of the Mean
MAPK: Mitogen-activated protein kinase
DDP: Cisplatin
PBS: Phosphate-buffered saline
OXPHOS: Mitochondrial Oxidative Phosphorylation System
ERK 1/2: Extracellular signal-regulated kinases 1/2
PDX: Patient-derived xenograft
PD-L1: Programmed death-ligand 1
